# Comparison of monoamine oxidase inhibition by cigarettes and modified risk tobacco products

**DOI:** 10.1016/j.toxrep.2019.11.008

**Published:** 2019-11-13

**Authors:** Marco van der Toorn, Kyoko Koshibu, Walter K. Schlage, Shoaib Majeed, Pavel Pospisil, Julia Hoeng, Manuel C. Peitsch

**Affiliations:** a Department of Systems Toxicology, PMI R&D, Philip Morris Products S.A., Quai Jeanrenaud 5, CH-2000, Neuchâtel, Switzerland; b Biology Consultant, Max-Baermann-Str. 21, 51429, Bergisch Gladbach, Germany

**Keywords:** pMRTP, potential modified risk tobacco products, MAO, monoamine oxidases, 3R4F, reference cigarette, THS, Tobacco Heating System, *MESH*, electronic cigarette, PREP, potential reduced exposure products, CS, cigarette smoke, TPM, total particulate matter (TPM), PMI, Philip Morris International, cDNA, complementary DNA, Km, Michaelis constant, CRP, CORESTA Reference Product, GCW, General Classic White, GVP, gas–vapor phase, RT, room temperature, GC, gas chromatography, FID, flame ionization detection, DMSO, dimethyl sulfoxide, PBS, phosphate-buffered saline, IC_50_, half maximal inhibitory concentrations, Ki, Inhibition Constant, Monoamine oxidase, Harm reduction, Tobacco heating system, E-cigarettes, Snus

## Abstract

The adverse effects of cigarette smoking are well documented, and the two main strategies for reducing smoking prevalence are prevention of smoking initiation and promotion of smoking cessation. More recently, a third and complementary avenue, tobacco harm reduction has emerged, which is aimed to reduce the burden of smoking-related diseases. This has been enabled by the development of novel products such as electronic cigarettes (e-cigarettes) and heated tobacco products, designed to deliver nicotine with significantly reduced levels of the toxicants that are emitted by cigarettes. Several potential modified risk tobacco products (pMRTP) have been reported to emit significantly less toxicants than cigarettes and significantly reduce toxicant exposure in smokers who switch completely to such products. These are two prerequisites for pMRTPs to reduce harm and the risk of smoking-related disease. However, concerns remain regarding the addictive nature of these products. Smoking addiction is a complex phenomenon involving multiple pharmacological and non-pharmacological factors. Although the main pharmacological substance associated with smoking addiction is nicotine, accumulating evidence suggests that nicotine mostly acts as a primary reinforcer and that other factors are involved in establishing smoking addiction. Inhibition of monoamine oxidases (MAO)—mammalian flavoenzymes with a central role in neurotransmitter metabolism—has also been suggested to be involved in this process. Therefore, we aimed to comparatively investigate the ability of several types of pMRTPs and cigarette smoke (3R4F) to inhibit MAO activity. The results showed that the heated tobacco product Tobacco Heating System (THS) 2.2 and the *MESH 1.1* e-cigarette possessed no MAO inhibitory activity while 3R4F significantly inhibits the levels of MAO activity (3R4F MAO-A and B; > 2 μM nicotine). Snus products have similar inhibition profiles as 3R4F but for larger nicotine concentrations (snus MAO-A; ∼68-fold, snus MAO-B; ∼23-fold higher compared to 3R4F). These observations were confirmed by analytical datasets of potential MAO inhibitors emitted by these products. In conclusion, we have demonstrated that specific pMRTPs, namely THS 2.2 and *MESH 1.1*, have a significantly lower MAO-inhibitory activity than 3R4F. These findings provide a basis for further investigation of the role of MAO inhibitors in cigarette addiction as well as the implications of the findings for abuse liability of pMRTPs in comparison with cigarettes.

## Highlights

• Aerosol fractions from heated tobacco and e-cigarettes do not inhibit MAO activity.
• Smoke fractions from burned (cigarette) and extracts from smokeless (snus) tobacco inhibit MAO activity.
• MAO inhibition in the different products is independent from their nicotine concentrations.

## Graphical abstract

**Figure:**
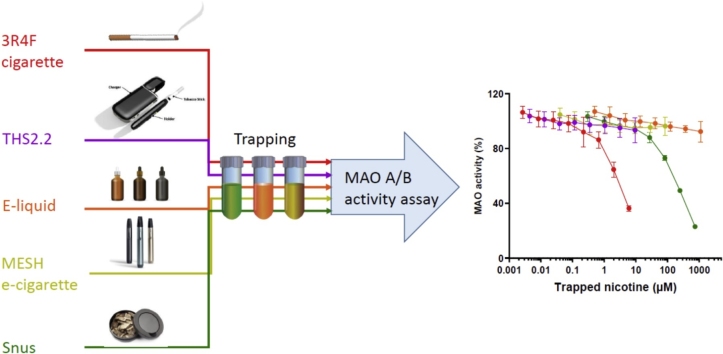

## 1 Introduction

Cigarette smoking has been causally linked to major preventable diseases, morbidity, and mortality worldwide. The most effective way to reduce the adverse public health impact of tobacco products is to reduce their use by preventing smoking initiation, promoting smoking cessation, and/or reducing the toxicity of the products [1,2]. Worldwide smoking/tobacco control policies have contributed to a considerable decrease in smoking prevalence, resulting in as much as a 50 % decrease in the USA and UK [3,4]. However, smoking dependence and adaptation to smoking habits are still prevalent, particularly among heavy smokers, whose attempts to quit often fail (even when supported by nicotine-replacement therapy) and for whom the success rate of long-term (>1 year) abstinence is typically around 5 % or less [5,1]. The harm caused by tobacco products is a result of frequent exposure to the toxic byproducts of combustion rather than nicotine (the main psychoactive chemical in tobacco products) [[6], [7], [8],^4^]. Therefore, the Institute of Medicine has further developed and defined the concept of tobacco harm reduction and suggested a regulatory and scientific framework for developing less hazardous tobacco products with some characteristics of cigarettes, termed potential reduced exposure products (PREP) or modified-risk tobacco products (MRTP) [9,10]. The spectrum of potential MRTPs (pMRTPs) includes heated tobacco products, electronic cigarettes (e-cigarettes), and oral smokeless tobacco, such as Swedish snus. All these products are designed to deliver nicotine and flavors with significantly reduced levels of accompanying toxicants.

Although smoking-related diseases are mainly caused by the toxic byproducts of combustion contained in cigarette smoke (CS) [7,8,4], nicotine and other pharmacological and non-pharmacological factors play a distinct role in the abuse liability of nicotine-containing products [7,11,4]. Among other psychopharmacologically active compounds, a variety of substances in tobacco leaves and tobacco smoke that inhibit brain monoamine oxidase (MAO) activity have been investigated for their role in dependence-inducing effects [12]. MAOs are mammalian flavoenzymes bound to the outer mitochondrial membrane that play a central role in neurotransmitter metabolism [13]. The interest in investigating the role of MAO in smoking dependence stems from clinical studies demonstrating that both MAO-A and MAO-B activities are inhibited in the brain of smokers (relative to non-smokers) [[14], [15], [16]] and in animals exposed to CS [12,[17], [18], [19]]. In fact, evidence suggests that MAO inhibition can potentiate the reinforcing effects of systemically administered nicotine in animal models [12,20,21]. However, a correlation between the exact substances responsible for MAO inhibition in adult smokers and their contribution to smoking addiction has not been clearly demonstrated in humans [12]. In pMRTPs, many tobacco or tobacco smoke constituents (except nicotine) are reduced in concentration or completely removed, including potential MAO inhibitors. While nicotine itself has no MAO-inhibitory potential even at concentrations far above the plasma nicotine concentrations in smokers [17,22] a synergistic or additive MAO-inhibition potential of the remaining compounds cannot be excluded [12]. CS total particulate matter (TPM) and methanolic CS extracts have been reported to display MAO-inhibitory activity [[23], [24], [25]]. However, potential MAO inhibition by pMRTPs such as e-cigarettes, heated tobacco products, and smokeless tobacco has not been reported so far. Therefore, we chose to compare the inhibitory potential of various pMRTPs (i.e., an e-cigarette e-liquid; extracts from reference and commercial brands of Swedish snus; and three types of aerosol fractions from the heated tobacco product Tobacco Heating System (THS) 2.2 and e-cigarette (*MESH* 1.1) and the corresponding fractions of CS) on the activity of recombinant human MAO-A and MAO-B proteins using a two-step bioluminescent assay in order to elucidate potential additional factors that can differentiate these products and their risk of dependence.

## 2 Materials and methods

### 2.1 Materials

The MAO-Glo assay system (cat. no. V1402) was purchased from Promega (Dübendorf, Switzerland). The human MAOs used in this study were derived from insect cells infected with recombinant baculovirus containing complementary DNA (cDNA) inserts for human MAO-A and MAO-B (Sigma–Aldrich Chemie GmbH, Buchs, Switzerland cat. no. M7316 and M7441, respectively). M30 dihydrochloride and pargyline hydrochloride were purchased from Sigma–Aldrich.

### 2.2 MAO assay

The two-step bioluminescent assay was performed in Nunc white, 96-well, flat-bottom assay plates (Life Technologies Europe B.V., Zug, Switzerland). In the MAO reaction, recombinant MAO (MAO-A and MAO-B, 0.4 and 0.1 U/well of microsomal protein, respectively) was incubated with derivative of beetle luciferin ((4S)-4,5-dihydro-2-(6-hydroxybenzothiazolyl)-4-thiazolecarboxylic acid) substrate and sample/vehicle for 1 h at room temperature in a 50-μL reaction mixture. The beetle luciferin substrate concentrations were varied for determining the kinetic constants, but all subsequent experiments used the substrate at the Michaelis constant (Km) values of 20 μM and 3 μM (MAO-A and MAO-B, respectively). In the luciferin detection reaction, 50 μL of luciferin detection reagent (Promega) was added to the MAO reaction. After a 1-h incubation period, the luminescent signal was measured by using a Fluostar Omega 96 Microplate reader (BMG LABTECH GmbH, Ortenberg, Germany).

### 2.3 Reference cigarette 3R4F, THS 2.2, *MESH*1.1, snus, and e-liquid

Mainstream cigarette smoke was generated from reference research filtered cigarettes 3R4F, purchased from the University of Kentucky (Lexington, KY, USA; http://www.ca.uky.edu/refcig/). Test aerosol was generated from THS 2.2, a heat-not-burn product developed by Philip Morris International (PMI) and commercialized under the brand name *IQOS*®. THS 2.2 sticks require a THS 2.2 device, operated by inserting a stick into a tobacco stick holder, which heats the tobacco plug to generate an aerosol containing water, glycerin, nicotine, and tobacco flavors [26]. The THS 2.2 device includes a stick holder, a battery, electronics for control, a heating element, and a stick extractor [27]. THS 2.2 sticks and devices were provided by PMI (Neuchâtel, Switzerland). Prior to use in the study, 3R4F cigarettes and THS 2.2 sticks were conditioned in accordance with ISO Standard 3402 [28] for 7–21 days. Test aerosol from *MESH* Tobacco Harmony flavor caps was generated by using an e-cigarette device with *MESH* 1.1 technology (P4M3 generation 1.1, PMI), which maintains the temperature of the heater between 200–220℃ rather than vary it depending on puff strength. The *MESH* 1.1 device and caps were provided by PMI. Swedish snus extracts were obtained from the CORESTA Reference Product 1.1 (snus_CRP1.1, Tobacco Analytical Services Laboratory, North Carolina State University, Raleigh, NC, USA), General Classic White snus (snus_GCW, Swedish Match, Stockholm, Sweden), and TAXI regular (snus_TAXI, Philip Morris, South Africa).

### 2.4 Generation of smoke/aerosol, aqueous extracts, gas–vapor phase (GVP) extracts, and total particulate matter (TPM)

CS from 3R4F cigarettes was generated on a 20-port Borgwaldt smoking machine (Hamburg, Germany), and test aerosol from THS 2.2 was generated on a 30-port SM2000/P1 smoking machine (PMI, Neuchatel, Switzerland) in accordance with the Health Canada intense smoking protocol (puff volume, 55 mL; puff duration, 2 s; puff frequency, 2 min^−1^; 100 % blocking of filter ventilation holes for 3R4F) [29]. Test aerosol from *MESH* 1.1 was generated on a CETI8 Range smoking machine (CERULEAN, Milton Keynes, UK) in accordance with the modified Health Canada intense smoking protocol (puff volume, 55 mL; puff duration, 3 s; puff frequency, 2 min^−1^) [30].

Aqueous extracts were generated by bubbling aerosol or smoke through phosphate-buffered saline (PBS; 3R4F: 6 cigarettes/36 mL PBS, total puffs 61.7; THS 2.2: 10 sticks/40 mL PBS, total puffs 120; and *MESH* 1.1: 1 cap/10 mL PBS, total puffs 50) on ice. All aqueous extract solutions were further diluted in PBS to obtain final concentrations ranging from 0.01 to 0.5 puffs/mL. For collecting GVP extracts, aerosol or smoke was first passed through a Cambridge glass-fiber filter (Ref 8020 285 1; 44-mm diameter; Borgwaldt) before being bubbled through PBS (3R4F: 6 cigarettes/36 mL PBS, total puffs 61.7; THS 2.2: 10 sticks/40 mL PBS, total puffs 120; and *MESH* 1.1: 1 cap/10 mL PBS, total puffs 50) on ice. All GVP extract solutions were further diluted in PBS to obtain final concentrations ranging from 0.01 to 0.5 puffs/mL. TPM was collected on Cambridge glass-fiber filters (44-mm diameter; Borgwaldt). TPM samples from aerosol or mainstream smoke (3R4F: total puffs, 61.7; THS 2.2: total puffs, 120; and *MESH* 1.1: total puffs, 50) were collected and then extracted with an appropriate volume of dimethyl sulfoxide (DMSO) to a final concentration of 50 mg TPM/mL for 3R4F, THS 2.2, and *MESH* 1.1.

### 2.5 Snus_CRP1.1, snus_GCW, and snus_TAXI extracts

Snus packages were stored at −20℃. The snus boxes were placed at +4℃ for a minimum of 24 h prior to the experiments. One hour before extraction, the snus pouches were placed at room temperature (RT) for equilibration. Five snus pouches of each type were cut in half, and the pouch material and content (5 g) were extracted with 25 mL PBS (1/5 dilution) for 1 h at 37℃ under agitation (400 rpm). The extract was then centrifuged at 2400 rpm for 5 min at RT. The supernatant was subsequently centrifuged in a second centrifuge tube equipped with a filter cup (Labo Service Belgium bvba, Kontich, Belgium) protected with a glass-fiber filter pad (0.45-μm syringe filter, Fisher Scientific, Waltham, MA, USA) and centrifuged for 10 min at 2400 rpm. The centrifuged extract was loaded into a 10-mL glass syringe with a polytetrafluoroethylene head and filtered through a 0.22-μm pore-size syringe filter (Fisher Scientific; polyethersulfone membrane, 33-mm diameter, sterile). The snus preparations obtained were further diluted in PBS to obtain final concentrations ranging from 0.009–20 mg/mL.

### 2.6 E-liquid

E-liquid testing was performed by extracting e-liquid from *MESH* Tobacco Harmony flavor caps (PMI; 18 mg/mL nicotine). The e-liquid solution was further diluted in PBS to obtain final concentrations ranging from 0.00046 % to 1 %.

### 2.7 Nicotine analysis

For monitoring batch consistency, the nicotine content in whole smoke/aerosol aqueous extracts, GVP extracts (in PBS), TPM extracts (in DMSO) and snus extracts was determined (Table A2). After generation of the different fractions, an aliquot of 100 μL was transferred to a vial containing 900 μL n-butyl acetate and 0.1 % trimethylamine, with isoquinoline as the internal standard. Nicotine analysis was performed by gas chromatography (GC) with flame ionization detection (GC-FID) by using an Agilent 7890A GC system (Agilent Technologies; Basel, Switzerland) equipped with a standard flame ionization detector. The GC device was equipped with a J&W capillary column DB-5 (15 m ×0.25 mm ID fused silica; film thickness, 0.25 μm; Agilent Technologies). The GC inlet was maintained at 220℃ with a constant flow (1.4 mL/min) of ultrapure helium (Carbagas, Gümligen, Switzerland) as the carrier gas. Ultra-zero air (Carbagas) and ultrahigh-purity hydrogen (Carbagas) were used for the flame ionization detector. An injection split ratio of 1:50 was used for these analyses. The GC oven was an isotherm at 140℃ for 2.5 min. The total GC run time was 2.5 min. All GC-FID analyses were performed once.

### 2.8 Statistical analysis

The half maximal inhibitory concentrations (IC_50_) and 95 % confidence interval were analyzed by using Prism 8 for Windows (Graphpad Software, Inc., San Diego, USA).

## 3 Results

### 3.1 MAO-A inhibition

**Fig. 1:**
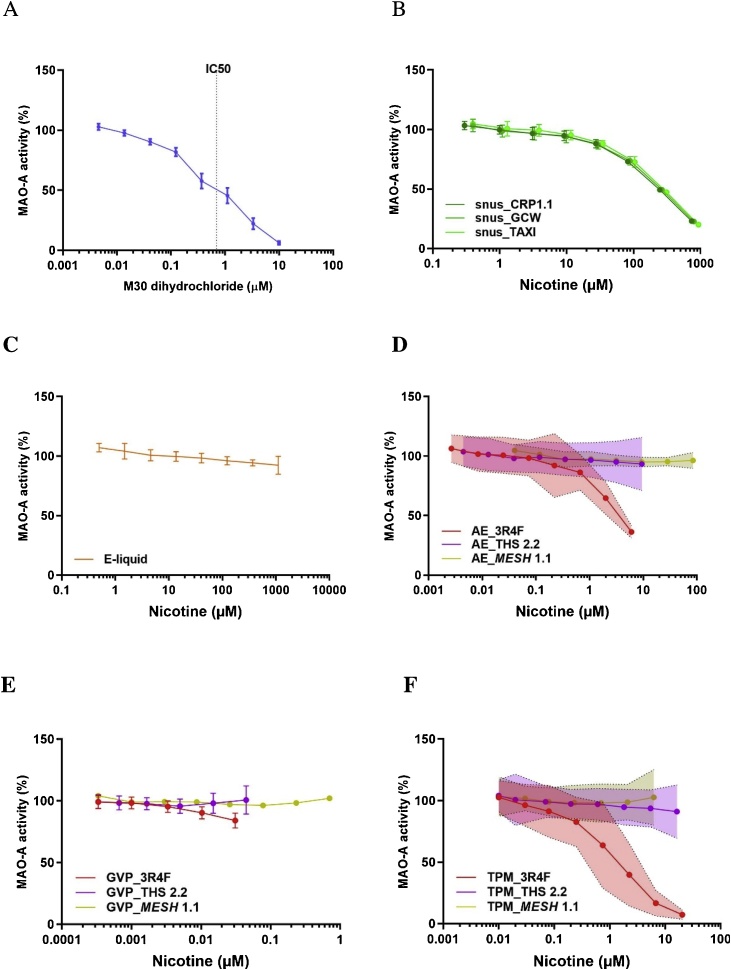
Effect of the reference compound and different nicotine-containing products on MAO-A activity. **A**) Reference compound M30 dihydrochloride; **B**) snus extracts from CRP1.1, GCW, and TAXI; **C**) e-liquid; **D**) AE from 3R4F, THS 2.2, and *MESH* 1.1; **E**) GVP extracts from 3R4F, THS 2.2, and *MESH* 1.1; and **F**) TPM from 3R4F, THS 2.2, and *MESH* 1.1. Data are expressed as mean ± standard deviation (A, B, C, E) or 95 % confidence interval (D, F) derived from three experiments. AE, aqueous extracts; GVP, gas–vapor phase; TPM, total particulate matter.

The positive control substance, M30, inhibited MAO-A activity in a dose-dependent manner, with an IC_50_ of 690 nM (Table A1; Fig. 1A). The three snus extracts also inhibited MAO-A activity in a dose-dependent manner. There was no difference between the reference product CRP1.1 and the two commercial products, GCW and TAXI, which had similar IC_50_ values (approximately 6 mg/mL, corresponding to 229–267 μM nicotine) (Table A1, Fig. 1B). The e-liquid from *MESH* 1.1 did not inhibit MAO-A activity in the tested concentration range (up to 1 % or 10 mg/mL, corresponding to approximately 1000 μM nicotine) (Fig. 1C). While only the aqueous extract and TPM of CS significantly inhibited MAO-A activity in a dose-dependent manner, none of the aerosol fractions of THS 2.2 or *MESH* 1.1 showed MAO-A inhibition (Fig. 1D, E, F). The IC_50_ values for the aqueous extract and TPM of CS were 0.09 puff/mL (corresponding to 3.6 μM nicotine) and 0.003 mg/mL (equal to approximately 0.015 puffs/mL, corresponding to 1.4 μM nicotine), respectively (Table A1).

### 3.2 MAO-B inhibition

**Fig. 2:**
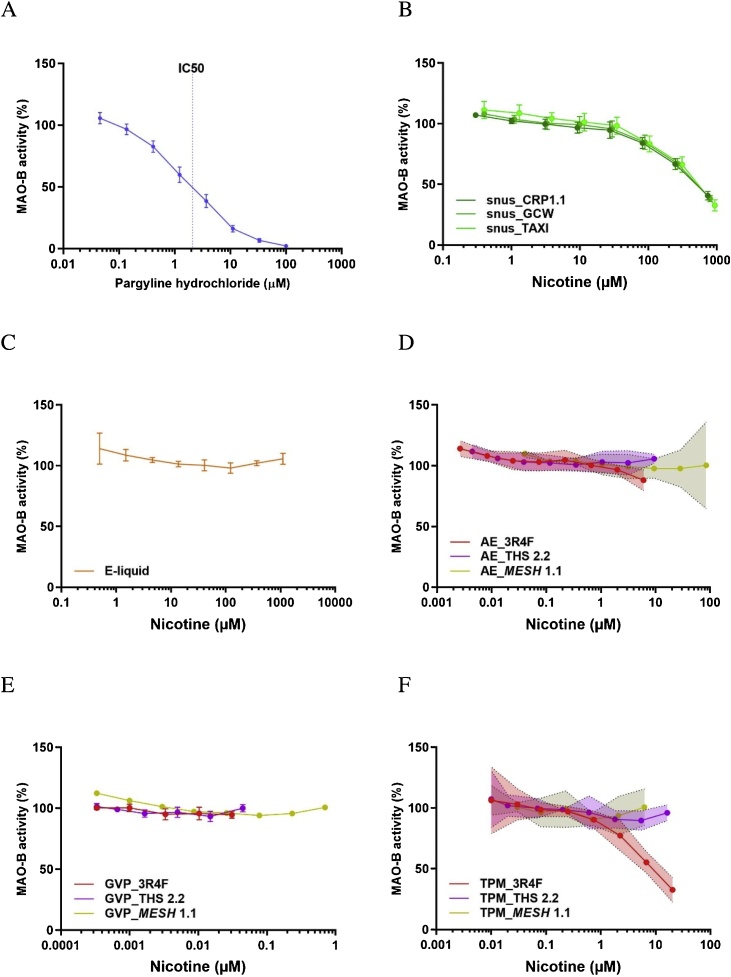
Effect of the reference compound and different nicotine-containing products on MAO-B activity. A) Reference compound pargyline hydrochloride; B) snus extracts from CRP1.1, GCW, and TAXI; C) e-liquid; D) AE from 3R4F, THS 2.2, and *MESH* 1.1; E) GVP extracts from 3R4F, THS 2.2, and *MESH* 1.1; and F) TPM from 3R4F, THS 2.2, and *MESH* 1.1. Data are expressed as mean ± standard deviation (A, B, C, E) or 95 % confidence interval (D, F) derived from three experiments. AE, aqueous extracts; GVP, gas–vapor phase; TPM, total particulate matter.

The positive control substance, pargyline, inhibited MAO-B activity in a dose-dependent manner, with an IC_50_ of 2.11 μM (Table A1; Fig. 2A). The three snus extracts also inhibited MAO-B activity in a dose-dependent manner, without any difference between the reference product CRP1.1 and the two commercial products, GCW and TAXI. The common IC_50_ value was in the range of 11–13 mg/mL, corresponding to 502–519 μM nicotine (Table A1; Fig. 2B). The e-liquid from *MESH* 1.1 did not inhibit MAO-B activity in the tested concentration range (up to 1 % or 10 mg/mL, corresponding to approximately 1000 μM nicotine) (Fig. 2C). While only the aqueous extract and the TPM of CS significantly inhibited MAO-B activity in a dose-dependent manner, none of the three aerosol fractions from THS 2.2 or *MESH* 1.1 showed MAO-B inhibition (Fig. 2D, E, F). The IC_50_ for the TPM of CS was 0.022 mg/mL (equal to approximately 0.1 puff/mL, corresponding to 8.9 μM nicotine) (Fig. 2F), while the aqueous extract of CS inhibited MAO-B activity at 0.2 puff/mL or above, with a predicted IC_50_ of 0.58 puff/mL, corresponding to 21.9 μM nicotine (Table A1; Fig. 2D). The GVP of CS showed no detectable MAO-B inhibition in the tested concentration range (up to 0.2 puff/mL, corresponding to 0.2 μM nicotine) (Fig. 2E).

It should be noted that the concentrations in Fig. 1, Fig. 2 were normalized for nicotine content. For absolute sample concentrations, see Table A1.

## 4 Discussion

Delivering nicotine without the harmful toxicants contained in CS has been proposed as an option for tobacco harm reduction [7,11,31,4]. Nicotine replacement therapies (NRTs) were introduced in the 1980s to help reduce the effects of the harmful substances in CS and aid smoking cessation. However, despite their potential to help smokers quit, NRTs met with moderate success: while some smaller studies reported 1-year quit rates around 50 %, e.g., [32], numerous larger studies and meta-analyses reported long-term quit rates of 25 % and less [[33], [34], [35]]. pMRTPs, on the other hand, are thought to provide an acceptable alternative to cigarettes by simulating some of the characteristics of cigarettes, such as the use of an inhalation device—which inevitably improves, for example, the pharmacokinetics of nicotine uptake—as well as greater physical appeal to smokers compared with NRTs. In fact, in a recent randomized trial reported by Hajek et al., the 1-year abstinence rate was 18.0 % in the e-cigarette group compared to 9.9 % in the nicotine-replacement group (relative risk, 1.83; 95 % confidence interval [CI], 1.30–2.58; P < 0.001), when accompanied by behavioral support [36]. In addition, among participants with 1-year abstinence, 80 % of the participants in the e-cigarette group used their assigned product at 52 weeks compared to only 9 % in the nicotine-replacement group, indicating better acceptance of e-cigarette as a replacement for cigarette smoking compared to NRT. However, apart from the reduced harm owing to lower exposure to CS toxicants, concerns related to the risk of developing dependence on these products remain.

Pharmacologically, the critical factor in smoking addiction as well as dependence on nicotine-containing products is the dose and rate of nicotine delivery, which essentially makes the products that rapidly deliver high doses of nicotine more addictive than those that slowly deliver nicotine at much lower doses [1,4]. Although nicotine has generally been accepted as the main reinforcer of smoking addiction or dependence, concerns have been raised regarding other CS and tobacco constituents, including MAO inhibitors [12,4].

The results obtained in this study confirm the previously reported inhibitory activity of CS TPM on both forms of human MAO [[23], [24], [25]], while CS GVP shows no inhibitory activity. Interestingly, a weak MAO-A-inhibitory effect was also observed with the aqueous extract of whole CS (which mostly contains GVP constituents and less than 10 % of TPM constituents trapped by our standardized trapping method). In contrast, none of the aerosol fractions from THS 2.2 or *MESH* 1.1 inhibited MAO activity over the wide concentration ranges tested. Given the nicotine concentrations present in these fractions, the effect of nicotine can be excluded, which is in agreement with the findings of a previous review article (Tables A1 & A2) [20]. However, the aqueous extracts of all three samples of Swedish snus consistently exhibited robust MAO inhibition. Therefore, we hypothesize that at least some of the MAO inhibitors are already present in processed tobacco leaves (snus), but they are not transferred to the aerosol of THS 2.2 when the tobacco is only heated.

### 4.1 Presence of MAO inhibitors in tobacco plant, CS, and THS 2.2 aerosol

The difference in MAO-inhibitory activity among various pMRTPs and CS may be attributed to the distinct distribution of MAO-inhibiting substances in the different products. Chemical substances that are present in either the tobacco plant or CS (or in both) have been summarized and published by Rodgman and Perfetti [37]. This list has been used to identify compounds known to be MAO inhibitors in CS from 3R4F and in aerosol from THS 2.2 [38]. Their concentrations in each item (cigarette for CS and sticks for THS 2.2 aerosol) were identified from latest reports [26,39], and their MAO-inhibitory activity was determined by using BindingDB (bindingdb.org) (Table 1). The corresponding data are not yet available for snus extracts and e-cigarette liquid and aerosol.

**Table 1:** Inhibitors of MAOs in tobacco leaves and tobacco smoke.

Compound name	CAS number	Unit	3R4F	THS 2.2	Presence in tobacco plant (T) or CS (S)	MAO inhibition, comment
Acetaldehyde	75-07-0	μg/item	1555	219	S	via condensation products with biogenic amines [40]
Harman	486-84-0	μg/item	25.473	0.278	T, S	MAO-A: Ki = 55.5 nM MAO-B: Ki = 320 nM
Norharman	244-63-3	μg/item	43.877	3.201	T, S	MAO-A: Ki = 1.2 μM; 4.7 μM MAO-B: Ki = 1.12 μM
Nornicotine	5746-86-1	μg/item	22.117	0.32	T, S	[41]
Anatabine	2743-90-0	μg/item	6.218	2.165	T, S	[41]
Anabasine	13078-04-1	μg/item	1.030	0.608	T, S	[41]
2-Naphthylamine	91-59-8	ng/item	11.0	0.046	S	MAO-A: Ki = 52 μM MAO-B: Ki = 40.2 μM
2,3,6-Trimethyl -1,4-naphthoquinone	20490-42-0	μg/item	1.299	0.064	T, S	MAO-A: Ki = 3 μM MAO-B: Ki = 6 μM
*trans*-*trans*-farnesol	106-28-5	μg/item	4.5	0.18	T, S	MAO-B: Ki = 800 nM MAO-B murine: Ki = 2.4 μM
Farnesylacetone	1117-52-8	μg/item	8.815	3.802	T, S	[42,43]
Nitric monoxide	10102-43-9	μg/item	491	16.8	T, S	[43]

Acetaldehyde—a major non-nicotine component of CS—has been suggested to contribute to smoking dependence [44,45]. It enhances nicotine self-administration in rats, most probably by an indirect mechanism of MAO inhibition – that is, via formation of condensation products of acetaldehyde and biogenic amines [such as harman] or tetrahydroisoquinolines [including salsolinol] in CS and/or in vivo environments [40,46,47]. Salsolinol and harmans have been found to be rewarding in rat behavioral studies [40,44,48,49]. However, free acetaldehyde is probably not responsible for the MAO inhibition observed in our present study, because it is not a significant constituent of snus extracts or CS TPM, while the GVP of CS (which contains high concentrations of acetaldehyde) had no inhibitory effect in the present in vitro enzyme assay; a study of the potential endogenous formation of inhibitory adducts in vivo was beyond the scope of this study.

The β-carbolines, harman and norharman, have been suggested as candidate tobacco-derived MAO inhibitors [23,40,46,50,51] (Table 1). They have been reported to contribute to a minimum of approximately 30 % of the MAO-inhibitory effect in the brain of smokers [52,53]. However, more recent investigations have indicated that harman and norharman provide only a moderate contribution to the total MAO-A-inhibitory activity of tobacco smoke, perhaps less than 10 % in vitro [54]. Harman and norharman were determined to be present in THS 2.2 aerosol at significantly lower (92- and 14-fold lower, respectively) concentrations than in 3R4F smoke.

Tobacco alkaloids other than nicotine—such as nornicotine, anatabine, and anabasine—are also directly rewarding in rats, possibly via inhibition of MAOs [41]. In contrast, according to Smith et al., a mixture of five minor alkaloids (nornicotine, anatabine, anabasine, cotinine, and myosmine), two beta-carbolines (harman and norharman), and acetaldehyde at smoking-relevant concentrations did not alter the primary reinforcing effects of nicotine in rats, while pharmacological MAO inhibitors did [21]. Nevertheless, as shown in Table 1, a recent analysis of nornicotine, anatabine, and anabasine [39] shows that these three alkaloids are present at low μg/cigarette concentrations in 3R4F smoke and in even lower concentrations in THS 2.2 aerosol. Nornicotine, anatabine, and anabasine were measured in THS 2.2 aerosol at approximately 70-, 2.8-, and 1.7-fold lower concentrations than in CS, respectively (Table 1), which suggests even lower potential MAO-inhibitor-induced effects of THS 2.2 on nicotine reinforcement.

Additional MAO inhibitors reported to be present in CS are 2-naphthylamine [55] and diethylnitrosamine [55,56]. 2-naphthylamine has been detected in CS (11.0 ng/cigarette) and in trace amounts in the aerosol from THS 2.2 (0.046 ng/stick). Given its low abundance and weak inhibitory activity (MAO-A: Ki = 52 μM; MAO-B: Ki = 40.2 μM), it is not likely to make a significant contribution to the observed MAO-inhibitory effect of CS TPM. Although diethylnitrosamine is listed as being present in tobacco plant and smoke, none of the product characterization studies indicate its detectable presence in 3R4F or THS 2.2.

A benzoquinone—2,3,6-trimethyl-1,4-naphthoquinone—with weak MAO-inhibiting activity (Ki = 3–6 μM) has been isolated from tobacco leaves and CS [57,58]. Our previous analysis identified the compound in CS (1.3 μg/cigarette) and at a 20-fold lower concentration in THS aerosol (Table 1). These concentrations were in agreement with the observed MAO-inhibitory activity of CS fractions. Moreover, MAO-inhibitory compounds farnesylacetone [42] and trans,trans-farnesol [24] have been reported to be present in both tobacco leaves and CS. Our analytical data indicated the presence of trans,trans-farnesol in CS (4.5 μg/cigarette) and at a 25-fold lower concentration in THS aerosol as well as the presence of farnesylacetone in CS (2.58 μg/cigarette) and at an 8-fold lower concentration in THS aerosol. These concentrations are in agreement with the observed MAO-inhibitory activity of the CS fractions.

Finally, nitric monoxide (NO)—a major component of CS GVP (reaching ppm levels in CS)—possesses MAO-inhibitory activity, as indicated by the finding that the NO donor *S*-nitroso-*N*-acetylpenicillamine (SNAP) can effectively inhibit MAO activity in the range of 0.4–40 μM [43]. In our present study, the GVP of CS did not exhibit MAO inhibition. The NO concentrations in CS versus those in THS 2.2 are presented in Table 1 [26], which indicates a 30-fold lower presence in THS 2.2 aerosol.

### 4.2 Future perspectives

Smoking addiction is a complex phenomenon that is mediated by nicotine and other pharmacological elements such as MAO, but equally important is a wide spectrum of non-pharmacological elements, such as habit learning, sensory cues, and environmental and psychosocial factors [59], as evidenced by improved smoking cessation by pMRTPs [36]. In addition, there are factors that imposes a greater susceptibility for smoking addiction in individuals, including genetic and personality traits as well as psychological status or mental health [60,61]. Our findings presented here supports and augment these previous observations that smoking addiction is indeed, a complex phenomenon and that the abuse liability of pMRTPs will need to be further investigated. While the results we obtained provide in vitro evidence that the aerosols of THS 2.2 and *MESH* 1.1 do not inhibit MAO activity, the absence of such inhibitory activity needs to be confirmed by investigating whether smokers who have completely switched to such products show lower levels of brain MAO inhibition than cigarette smokers. This could be achieved by using a positron-emission tomography approach, as described earlier [16,62,63]. Furthermore, it will be necessary to investigate whether THS 2.2 and *MESH* 1.1 aerosol TPM are less addictive than CS TPM using a more holistic approach taking into account for the pharmacological and non-pharmacological aspects in rodent behavioral studies. Finally, human studies will be necessary to determine whether MAO inhibitors actually play a significant role in cigarette addiction and whether heated tobacco products and e-cigarettes are less addictive than cigarettes. A positive outcome of such studies would indicate that smokers who completely switch to such pMRTPs would be more likely to successfully quit nicotine consumption over time, where pMRTPs would represent an intermediate step down in their cessation journey.

## Funding

This study was funded by Philip Morris International.

## Disclosure

All authors are employees of Philip Morris International or worked for PMI R&D under contractual agreements. Philip Morris International is the sole source of funding and sponsor of this project.

## Declaration of Competing Interest

The authors report no declarations of interest.

## Figures and Tables

**Table A1:** IC50 values and nicotine concentrations of different test items for inhibition of recombinant human MAO-A and MAO-B.

Test Items	MAO-A IC_50_						MAO-B IC_50_					
	Concentration (μM)	AE (puff/mL)	GVP extract (puff/mL)	TPM (mg/mL)	Extract (mg/mL)	e-liquid (%)	Concentration (μM)	AE (puff/mL)	GVP extract (puff/mL)	TPM (mg/mL)	Extract (mg/mL)	e-liquid (%)
**3R4F**	X	0.09 (3.6μM nicotine)	inactive	0.003 (1.4μM nicotine)	X	X	X	0.58 (21.9μM nicotine)	inactive	0.022 (8.9μM nicotine)	X	X
**THS 2.2**	X	inactive	inactive	inactive	X	X	X	inactive	inactive	inactive	X	X
***MESH* 1.1**	X	inactive	inactive	inactive	X	X	X	inactive	inactive	inactive	X	X
**snus_CRP1.1**	X	X	X	X	6.16 (229μM nicotine)	X	X	X	X	X	13.51 (502μM nicotine)	X
**snus_GCW**	X	X	X	X	6.14 (246μM nicotine)	X	X	X	X	X	12.74 (511μM nicotine)	X
**snus_TAXI**	X	X	X	X	5.69 (267μM nicotine)	X	X	X	X	X	11.06 (519μM nicotine)	X
**e-liquid**	X	X	X	X	X	inactive	X	X	X	X	X	inactive
**M30 dihydrochloride**	0.69	X	X	X	X	X	X	X	X	X	X	X
**Pargyline hydrochloride**	X	X	X	X	X	X	2.11	X	X	X	X	X

Data are expressed as the mean value derived from three independent extractions; x = not tested. AE, aqueous extracts; GVP, gas–vapor phase; TPM, total particulate matter; THS, tobacco heating system.

**Table A2:** Nicotine concentrations in the test samples.

Test Items	Nicotine concentration (mg/mL)				
	AE	GVP extract	TPM	extract	e-liquid
**3R4F**	0.0102	0.0002	3.3056	x	x
**THS 2.2**	0.0154	0.0002	2.6516	x	x
***MESH* 1.1**	0.1382	0.0034	1.0148	x	x
**snus_CRP1.1**	x	x	x	1.2046	x
**snus_GCW**	x	x	x	1.3016	x
**snus_TAXI**	x	x	x	1.5221	x
**e-liquid**	x	x	x	x	18

Data are expressed as the mean value derived from three independent extractions; x = not tested. AE, aqueous extracts; GVP, gas–vapor phase; TPM, total particulate matter; THS, tobacco heating system.
